# A survey of MRSA infection prevention practices amongst US burn centers

**DOI:** 10.1017/ash.2026.10873

**Published:** 2026-09-29

**Authors:** Natalie Mackow, Karen Brust, David Jay Weber, Werner Bischoff, Geeta Sood, Sara Karaba, Mohamed Yassin, Sheetal Kandiah, Jyoti Somani, Supriya Narasimhan, Larissa Pisney, Michelle Doll, Donald Chen, Lilian Abbo, Kelli M. Williams, Madhuri M. Sopirala

**Affiliations:** 1 Stony Brook University Renaissance School of Medicine, USA; 2 University of Iowa Health Care, USA; 3 The University of North Carolina at Chapel Hill School of Medicine, USA; 4 Wake Forest University School of Medicine, USA; 5 Johns Hopkins School of Medicine: The Johns Hopkins University School of Medicin, USA; 6 University of Pittsburgh School of Medicine, USA; 7 Emory University School of Medicine, USA; 8 University of Miami Miller School of Medicine: University of Miami School of Med, USA; 9 Santa Clara Valley Medical Center, USA; 10 University of Colorado School of Medicine: University of Colorado Anschutz Medic, USA; 11 VCU Health: Virginia Commonwealth University Health System, USA; 12 Westchester Medical Center, USA; 13 University of Cincinnati College of Medicine, USA; 14 Infectious Diseases/Internal Medicine, UT Southwestern: The University of Texas Southwestern Medical Center, USA

## Abstract

Contemporary infection prevention practices for methicillin-resistant *Staphylococcus aureus* (MRSA) infections were surveyed across 12 mostly academic medical center burn units using the SHEA Research Network. There was variability in surveillance and decolonization practices, with 58% reporting active MRSA surveillance. More evidence-based data for burn MRSA infection prevention practices are needed.

## Introduction

Burn injury is a frequent source of morbidity and mortality worldwide with infections being a leading cause of death. Infectious complications with multidrug-resistant organisms are common in this population and associated with antibiotic exposure, prolonged hospitalizations, mechanical ventilation, and increased surgical procedures.^1^ In particular, methicillin-resistant *Staphylococcus aureus* (MRSA) is frequently identified in burn patients occurring early during hospitalization.^2^ Despite this, studies assessing the impact of MRSA infection on outcomes in burn patients are limited, with few studies focusing on MRSA infection prevention (IP) practices.

Currently, MRSA prevention practices vary by burn unit, as no standard guidelines exist. We sought to assess IP practices for MRSA across US burn centers.

## Methods

This pilot study used a survey developed by the Society for Healthcare Epidemiology of America (SHEA) Burn Infection Prevention and Control Special Interest Group to assess burn-unit infection prevention practices. The SHEA Research Network includes over 120 hospitals worldwide. Developed in Alchemer (Louisville, CO) with network staff, the survey was reviewed by experts and leadership for clarity, content validity, and flow. Invitations and reminders were emailed to 96 US facilities from April through June 2025. The survey is in the Supplement.

Responses were analyzed by facility. Those without burn units were excluded. Analyses used Microsoft Excel. Categorical variables are reported as counts and percentages and continuous variables as medians and ranges. Denominators vary with missing responses. Percentages for multiple-selection questions may not total 100%.

## Results

Survey invitations were distributed to 96 US facilities participating in the SHEA Research Network. Twenty-one facilities opted out, leaving 75 facilities in the response-rate denominator. Of these, 36 responded to the survey (48.0%). Among the responding facilities, 21 did not have a burn unit. Fifteen facilities reported having a burn unit; three did not provide additional survey responses. Therefore, 12 facilities with burn units were included in the analysis.

Characteristics of the responding facilities and burn units are summarized in Table 1. Most facilities had mixed burn units that included both intensive care unit (ICU) and non-intensive care unit beds. All burn units were managed by burn surgeons, and most were also managed by intensive care physicians.

**Table 1.** Characteristics of responding facilities and burn units Table 1 long description.

Characteristic	No./total No. (%)	Median (range)
**Hospital-level characteristics^a^**		
Academic medical center	10/10 (100)	–
**Total hospital beds**		
300–400	2/10 (20.0)	–
401–800	2/10 (20.0)	–
>800	6/10 (60.0)	–
**Pediatric beds at the facility**		
0	2/10 (20.0)	–
1–50	3/10 (30.0)	–
51–100	2/10 (20.0)	–
>100	3/10 (30.0)	–
Has an infection prevention program	10/10 (100)	–
Infection preventionists at the facility	–	7 (1–12)
Hospital epidemiologist staffing^b^	–	1.3 (0–1.8)
Has an antimicrobial stewardship program^c^	9/9 (100)	–
Antimicrobial stewardship staff^d^	–	3.2 (1–10)
**Burn-unit characteristics**		
Mixed ICU and non-ICU burn unit	11/12 (91.7)	–
Fixed ICU beds in the burn unit^e^	–	8 (4–13)
Fixed non-ICU beds in the burn unit^e^	–	10 (0–18)
New acute-care burn patients treated annually	–	325 (56–5,000)
**Physician specialties involved in burn-unit management^f^**		
Burn surgeons	12/12 (100)	
Intensive care physicians	10/12 (83.3)	–
Trauma surgeons	2/12 (16.7)	–
Plastic surgeons	1/12 (8.3)	–

ICU, intensive care unit.

^a^Ten facilities entered data for hospital-level characteristics.

^b^Nine facilities provided a hospital epidemiologist staffing value.

^c^Nine facilities selected “Yes”.

^d^Ten facilities provided antimicrobial stewardship staffing information.

^e^Eleven facilities responded.

^f^Multiple responses were allowed.

MRSA surveillance, decolonization, and wound-care practices are summarized in Table 2. Seven of 12 facilities performed active MRSA surveillance. Among facilities performing active surveillance, all screened patients at admission, and three also performed weekly screening. Most responding facilities reported decolonizing patients colonized or infected with MRSA with universal decolonization more common than targeted decolonization. Chlorhexidine baths and nasal mupirocin were the most reported products used for MRSA decolonization. Most facilities routinely performed chlorhexidine bathing for all admitted patients. Diluted chlorhexidine and pure hypochlorous acid wound solution were the most commonly reported products routinely used on burn wounds. The median MRSA bacteremia rate reported by nine facilities was 0.400 per 1,000 patient-days.

**Table 2.** MRSA surveillance, decolonization, and wound-care practices in responding burn units Table 2 long description.

	No./total No. (%)	Median (range)
**MRSA bacteremia rate**		
MRSA bacteremia NHSN LabID events per 1,000 patient-days during the preceding 12 months^a^	–	0.40 (0.00–2.64)
**Active MRSA surveillance practices**		
Performs active MRSA surveillance	7/12 (58.3)	–
Does not perform active MRSA surveillance	5/12 (41.7)	–
**Timing of screening among facilities performing active surveillance** ^b^		
At admission	7/7 (100)	–
Weekly	3/7 (42.9)	–
At transfer to the burn unit	2/7 (28.6)	–
At discharge	1/7 (14.3)	–
**Decolonization of patients colonized or infected with MRSA**		
Performs MRSA decolonization^c^	9/11 (81.8)	–
Does not perform MRSA decolonization^c^	2/11 (18.2)	–
**Decolonization strategy among facilities reporting an approach** ^d^		
Universal decolonization	6/8 (75.0)	–
Targeted decolonization	2/8 (25.0)	–
**Products used for MRSA decolonization^b,e^**		
Chlorhexidine baths	11/11 (100)	–
Nasal mupirocin	8/11 (72.7)	–
Nasal povidone-iodine	4/11 (36.4)	–
Alcohol-based nasal antiseptic	1/11 (9.1)	–
Oral care	1/11 (9.1)	–
**Routine chlorhexidine bathing policies in the burn unit^b^**		
Routinely performed for all admitted patients	10/12 (83.3)	–
Routinely performed for patients with central lines	3/12 (25.0)	–
Routinely performed for patients colonized or infected with MRSA	2/12 (16.7)	–
**Decolonization products routinely used on burn wounds^b^**		
Diluted chlorhexidine	8/11 (72.7)	–
Vashe wound solution (pure hypochlorous acid)	4/11 (36.4)	–
Undiluted or 2% chlorhexidine	2/11 (18.2)	–
Primaderm spray on skin	1/11 (9.1)	–

CHG, chlorhexidine; MRSA, methicillin-resistant *Staphylococcus aureus*; NHSN, National Healthcare Safety Network.

^a^Nine facilities provided both patient-day data and a numeric MRSA bacteremia count. Rates were calculated as the number of MRSA bacteremia NHSN LabID events per 1,000 patient-days.

^b^Multiple responses were permitted.

^c^One facility selected “Don’t know” and was excluded from the denominator. One facility selected “Other” and specified universal decolonization of all patients; this response was categorized as performing decolonization.

^d^Eight facilities reported a decolonization strategy. One free-text response specifying universal decolonization of all patients was categorized as universal decolonization.

^e^Eleven facilities selected at least one product in response to the MRSA-decolonization products question.

## Discussion

This pilot survey evaluated MRSA prevention practices in 12 US burn units through the SHEA Research Network. It demonstrated substantial variability in surveillance, decolonization, and wound-care practices. This variation may reflect the limited burn-specific evidence base and reliance on practices extrapolated from general ICU infection prevention practices.

Burn patients are highly susceptible to infection with multidrug-resistant organisms due to loss of the skin protective barrier, need for invasive devices and mechanical ventilation, repeated wound excision and grafting surgeries, antibiotic exposures, and prolonged hospitalizations.^1,3^ MRSA is one of the most frequently encountered pathogens in burn wounds. Infections caused by MRSA can be severe and associated with sepsis, surgical graft loss, prolonged hospital length of stay, and increased mortality, especially in high-percent total body surface area burns.^4^ Colonization is often associated with subsequent MRSA infection in this population.^5^ Thus, early identification and reduction of MRSA colonization to prevent infection is an important target in this population. In this study, the median MRSA bacteremia rate reported by nine facilities was 0.400 per 1,000 patient-days, with variation between centers. The factors contributing to this variation were not assessed.

Nasal decolonization, universal skin decolonization, routine surveillance, and multimodal MRSA IP bundles have been assessed, each in small, single-center studies but mostly in non-burn ICU populations.^6,7^ While these approaches may support reductions in MRSA transmission and infection, there is limited evidence to support each of these IP practices either separately or combined in burn patients.^8^ Burn patients notably differ from typical ICU patients due to open wounds and specialized wound-care practices, and evidence from ICU populations may not be fully generalizable to burn units.

The most common interventions utilized for decolonization, chlorhexidine baths and nasal mupirocin, mirror strategies utilized in ICU infection prevention bundles. More research is needed about the frequency with which surveillance should occur and regarding the practice of decolonization. Finally, burn units also reported using different antiseptic agents during wound care, including diluted or undiluted chlorhexidine, pure hypochlorous acid, and specific wound cleanser sprays. These varied practices may reflect different institutional wound care protocols or surgeon preferences. There are limited data comparing these different wound care antiseptics specifically for MRSA prevention, although small studies exist comparing some products with sterile saline. Notably, chlorhexidine, which was used by many burn centers for open wounds, may be associated with cytotoxicity and chemical burns, although clinical data in burn wounds are limited.^9^ The successful application of universal decolonization with intra-nasal mupirocin and hypochlorous acid bathing in burn patients has been described in a single-center retrospective study.^10^ There is also a registered randomized controlled trial comparing sterile saline, chlorhexidine, and pure hypochlorous acid in children with burn wounds (NCT06941571).

Limitations of this survey-based study include response and sampling biases. Survey response was modest, and those who responded may reflect different medical center types or resources compared with those who did not. The responding institutions for which hospital type was reported included a select group of US academic medical centers. Only SRN-affiliated centers were surveyed, which may not capture a representative cohort of American Burn Association-verified burn centers. In addition, survey responses were self-reported and were not independently validated. Responses varied by survey item, resulting in changing denominators. The survey assessed reported practices but did not measure adherence to these practices or determine whether specific practices were associated with lower MRSA bacteremia rates or other patient outcomes.

Burn units currently rely on institution-specific MRSA prevention strategies, and a lack of standardized guidance may lead to varied prevention practices. Therefore, prospective, multicenter studies are needed to assess these variations and standardize best practices. These studies should inform guidance about the utility of active versus passive surveillance, universal versus targeted decolonization, and the effectiveness of antiseptic wound care agents for prevention of MRSA infections.

## Supporting information

10.1017/ash.2026.10873.sm001 Mackow et al. supplementary material Mackow et al. supplementary material

## Supplementary material

The supplementary material for this article can be found at https://doi.org/10.1017/ash.2026.10873.

## Table 1. Long description

The table summarizes characteristics of responding facilities and burn units. It has 3 sections and 12 rows and 3 columns. The first section, Hospital-level characteristics, includes rows for Academic medical center, Total hospital beds, Pediatric beds at the facility, Has an infection prevention program, Infection preventionists at the facility, Hospital epidemiologist staffing, Has an antimicrobial stewardship program, and Antimicrobial stewardship staff. The second section, Burn-unit characteristics, includes rows for Mixed ICU and non-ICU burn unit, Fixed ICU beds in the burn unit, Fixed non-ICU beds in the burn unit, and New acute-care burn patients treated annually. The third section, Physician specialties involved in burn-unit management, includes rows for Burn surgeons, Intensive care physicians, Trauma surgeons, and Plastic surgeons. Each row provides the number and percentage of facilities or units with the specified characteristic, along with the median and range where applicable.

Navigate back to Table 1..

## Table 2. Long description

A table with 12 rows and 3 columns summarizing MRSA surveillance, decolonization, and wound-care practices in burn units. The columns are labeled ‘No./total No. (%)’, ‘Median (range)’, and the row labels include various practices and their corresponding data. Row 1: MRSA bacteremia rate, MRSA bacteremia NHSN LabID events per 1,000 patient-days during the preceding 12 months, 0.40 (0.00-2.64). Row 2: Performs active MRSA surveillance, 7/12 (58.3), -. Row 3: Does not perform active MRSA surveillance, 5/12 (41.7), -. Row 4: At admission, 7/7 (100), -. Row 5: Weekly, 3/7 (42.9), -. Row 6: At transfer to the burn unit, 2/7 (28.6), -. Row 7: At discharge, 1/7 (14.3), -. Row 8: Performs MRSA decolonization, 9/11 (81.8), -. Row 9: Does not perform MRSA decolonization, 2/11 (18.2), -. Row 10: Universal decolonization, 6/8 (75.0), -. Row 11: Targeted decolonization, 2/8 (25.0), -. Row 12: Chlorhexidine baths, 11/11 (100), -. Row 13: Nasal mupirocin, 8/11 (72.7), -. Row 14: Nasal povidone-iodine, 4/11 (36.4), -. Row 15: Alcohol-based nasal antiseptic, 1/11 (9.1), -. Row 16: Oral care, 1/11 (9.1), -. Row 17: Routinely performed for all admitted patients, 10/12 (83.3), -. Row 18: Routinely performed for patients with central lines, 3/12 (25.0), -. Row 19: Routinely performed for patients colonized or infected with MRSA, 2/12 (16.7), -. Row 20: Diluted chlorhexidine, 8/11 (72.7), -. Row 21: Vashe wound solution (pure hypochlorous acid), 4/11 (36.4), -. Row 22: Undiluted or 2 percent chlorhexidine, 2/11 (18.2), -. Row 23: Primaderm spray on skin, 1/11 (9.1), -.

Navigate back to Table 2..
