# Supplementary material for: A survey of MRSA infection prevention practices amongst US burn centers
Source: Antimicrob Steward Healthc Epidemiol. 2026 Sep 29;6(1):e302. doi: 10.1017/ash.2026.10873 (PMC13760382; doi:10.1017/ash.2026.10873)
Supplement: Supplementary file 1 [file S2732494X26108730sup001.pdf]

SRN Study: Infection Prevention Practices in Burn Units

Healthcare Epidemiology/Infection Prevention Environmental-Surface Sampling Practices

ID 186

1. SRN ID: *

ID 187

2. System SRN ID (if applicable)

ID 195

3. Do you have a Burn Unit? *

- ○ No, I will opt out of the survey
- ○ Yes – please continue to question #4

Demographics

Page entry logic:
This page will show when: #3 Question "Do you have a Burn Unit?" is one of the following answers ("Yes – please continue to question #4")

VALIDATION Max. answers = 3 *(if answered)*

ID 2

4. What best describes your facility (max 3):

- ☐ Academic medical center

- ☐ Acute care - adult and pediatric
- ☐ Acute care - adult only
- ☐ Acute care - pediatric only
- ☐ Community hospital with academic affiliation
- ☐ Community hospital, no academic affiliation
- ☐ Correctional facility
- ☐ Critical access hospital
- ☐ Federal military hospital
- ☐ Federal non-military hospital
- ☐ Freestanding children's hospital
- ☐ Healthcare system leadership/headquarters
- ☐ Home care
- ☐ Independent consultant
- ☐ Indian Health Service
- ☐ Long-term acute care hospital
- ☐ Memory care residential facility
- ☐ Mental health facility
- ☐ Nursing home
- ☐ Outpatient
- ☐ Public health
- ☐ Rehabilitation
- ☐ Rural hospital
- ☐ Teaching
- ☐ Veterans Affairs
- ☐ Other ____________

3

5. Location of facility (state/province):

188

6. Location of facility (country):

4

7. Total beds in the facility:

- 0-50
- 51-100
- 101-300
- 301-500
- 501-1,000
- >1,000

ID 5

8. Total ICUs in facility:

- 1-3
- 4-6
- 7-9
- 10-12
- 13-15
- >15

ID 6

9. Is your facility part of a larger healthcare system?

VALIDATION Max. answers = 3 *(if answered)*

ID 7

10. What best describes your primary role? (max 3)

- ☐ Administration
- ☐ Antibiotic stewardship
- ☐ Basic research
- ☐ Clinical microbiology
- ☐ Clinical research
- ☐ Diagnostic stewardship
- ☐ Geriatrics
- ☐ Hospital administration/leadership
- ☐ Hospital epidemiology
- ☐ Infectious diseases physician
- ☐ Implementation science
- ☐ Infection prevention
- ☐ Internal medicine
- ☐ Laboratory medicine
- ☐ Pathology
- ☐ Patient care
- ☐ Pediatrics
- ☐ Performance improvement
- ☐ Public health
- ☐ Quality improvement
- ☐ Research methodology
- ☐ Teaching/education
- ☐ Transplant medicine
- ☐ Other ______

Burn Unit Details

Page entry logic:
This page will show when: #3 Question "Do you have a Burn Unit?" is one of the following answers ("Yes – please continue to question #4")

197

11. Is your burn unit a mixed care unit (includes both ICU and non-ICU status patients)?

- Yes
- No

198

12. How many fixed ICU beds does your burn unit have?

199

13. How many fixed non-ICU beds does your unit have?

200

14. On average, how many new acute care burn patients per year are treated in your burn unit?

201

15. Type of physicians that manage the unit (check all that apply):

- ☐ Burn surgeons
- ☐ Intensive care physicians
- ☐ Other - specify

MRSA Surveillance

Page entry logic:
This page will show when: #3 Question "Do you have a Burn Unit?" is one of the following answers ("Yes – please continue to question #4")

203

16. Does your unit perform active surveillance for MRSA (test for colonization of MRSA using nares swabs and/or other areas such as axilla or groin) at regular intervals during admission? (please select all that apply)

- ☐ Not done
- ☐ At time of admission
- ☐ Weekly
- ☐ Time of transfer to burn unit
- ☐ Time of discharge
- ☐ Other - please explain

MRSA Decolonization

Page entry logic:
This page will show when: #3 Question "Do you have a Burn Unit?" is one of the following answers ("Yes – please continue to question #4")

LOGIC Show/hide trigger exists.

ID 204

17. Does your burn unit perform decolonization for MRSA (such as CHG bathing treatment or nasal mupirocin or nasal povidone iodine)?

- Yes
- No

LOGIC Hidden unless: #17 Question "Does your burn unit perform decolonization for MRSA (such as CHG bathing treatment or nasal mupirocin or nasal povidone iodine)?" is one of the following answers ("Yes")

ID 205

18. If you answered yes to question , do you perform:

- Targeted (defined as only for patients who tested positive for MRSA either by clinical culture or active surveillance testing for colonization)
- Universal decolonization (defined as “for all patients regardless of whether they are infected or colonized”)?

ID 206

19. What products does your unit use for MRSA decolonization? (select all that apply)

- Chlorhexidine (CHG) baths
- Nasal mupirocin
- Nasal povidone iodine
- Other - please specify

207

20. Do you routinely perform chlorhexidine (CHG) bathing? If yes, check all that apply:

- ☐ On all patients on your burn unit
- ☐ On central line patients
- ☐ On MRSA colonized or infected patients

208

21. Which of the following decolonization products do you routinely use on burn wounds?

- ○ Diluted chlorhexidine (CHG)
- ○ Vashe (Pure hypochlorous acid)
- ○ Other - please specify

Personal Protective Equipment

Page entry logic:
This page will show when: #3 Question "Do you have a Burn Unit?" is one of the following answers ("Yes – please continue to question #4")

209

22. What dictates the required use of gown and gloves as barrier protection on your burn unit? (select all that apply)

- ☐ Any time a patient room is entered
- ☐ Bathing
- ☐ Dressing wounds
- ☐ Based on burn percentage (total body surface area)
- ☐ MRSA colonized patient
- ☐ MRSA infected patient
- ☐ Infection or colonization with other multidrug resistant organism - please specify
- ☐ Other - write in

MRSA Bloodstream Infections

Page entry logic:
This page will show when: #3 Question "Do you have a Burn Unit?" is one of the following answers ("Yes – please continue to question #4")

210

23. In the last 12 months, what was the number per patient days of documented hospital-onset MRSA bacteremia your burn unit (per NHSN MRSA Bacteremia LabID Events surveilled by your infection prevention team)?

211

24. In the last 12 months, what is the number of patient days in your burn unit?

Thank You!

1

Thank you for taking our survey. Your response is very important to us.
